# Weak-Periodic Stochastic Resonance in a Parallel Array of Static Nonlinearities

**DOI:** 10.1371/journal.pone.0058507

**Published:** 2013-03-11

**Authors:** Yumei Ma, Fabing Duan, François Chapeau-Blondeau, Derek Abbott

**Affiliations:** 1 College of Automation Engineering, Qingdao University, Qingdao, People’s Republic of China; 2 Laboratoire d’Ingénierie des Systèmes Automatisés, Université d’Angers, Angers, France; 3 Centre for Biomedical Engineering and School of Electrical & Electronic Engineering, The University of Adelaide, Adelaide, Southern Australia, Australia; University of Maribor, Slovenia

## Abstract

This paper studies the output-input signal-to-noise ratio (SNR) gain of an uncoupled parallel array of static, yet arbitrary, nonlinear elements for transmitting a weak periodic signal in additive white noise. In the small-signal limit, an explicit expression for the SNR gain is derived. It serves to prove that the SNR gain is always a monotonically increasing function of the array size for any given nonlinearity and noisy environment. It also determines the SNR gain maximized by the locally optimal nonlinearity as the upper bound of the SNR gain achieved by an array of static nonlinear elements. With locally optimal nonlinearity, it is demonstrated that stochastic resonance cannot occur, i.e. adding internal noise into the array never improves the SNR gain. However, in an array of suboptimal but easily implemented threshold nonlinearities, we show the feasibility of situations where stochastic resonance occurs, and also the possibility of the SNR gain exceeding unity for a wide range of input noise distributions.

## Introduction

Stochastic resonance (SR) is a nonlinear phenomenon where the transmission of a coherent signal by certain nonlinear systems can be improved by the addition of noise [1]–[12]. The SR effect was initially observed in a bistable climate model driven by a subthreshold periodic input [1]–[4]. Then, this phenomenon attracted much attention in physics and biology [2], [5], [6], [8], [13], [14]. It is reported that SR occurs in peripheral [5], [6], [8], [13]–[16] and central [17]–[19] nervous systems, since the nervous systems implement, as a basis, complex dynamics that very often involve nonlinear processes, and commonly have to operate in environments containing noise, either of external or internal origins [8], [20]. The SR phenomenon can also be observed at a behavioral level, for instance, feeding paddle-fish [21], human posture stabilization [22], [23] and attention control [24]. Currently, the utilization of noise has become an optional and nontrivial strategy for statistical signal processing. It is noted that different static nonlinearities have been employed to exhibit the SR effect, for instance, the threshold nonlinearity [3], [4], [8], [25], the saturation nonlinearity [7], the power-law sensor [26], non-adjustable [27]–[29] or variable [30] detectors, estimators [30]–[35] and optimal processors [36]. By including nonlinear elements into an array, the array enhanced SR effect was observed by tuning the array noise level and the coupling strength [37], [38]. Moreover, in the generic model of an uncoupled parallel array of static nonlinearities, some significant SR effects, e.g. SR without tuning [39], suprathreshold SR [40] and array SR [26], were subsequently reported. The constructive role of internal noise is adequately reappraised for improving the performance of the array of nonlinearities [31]–[34], [41]–[44]. Recently, the SR effect has been further shown with new characteristics in complex network topologies, such as small-world networks and scale-free networks [45]–[52]. Particularly, the influence of network architectures, as well as the non-zero noise level, on SR is recognized [45]–[52]. It is interesting to note that these related studies in general also provide evidence that, besides an optimal noise intensity, an optimal network configuration exists, at which the best system response can be obtained [49]–[52].

There has been considerable interest in the amplification of the signal-to-noise ratio (SNR) of a periodic signal by exploiting the SR effect [2], [8], [53]–[57]. From the viewpoint of the gain behavior, i.e., the SNR at the output divided by that at the input, the primary issue of a gain exceeding unity has been found for suprathreshold input signals [54]–[57]. However, most previous SR studies involved a fixed nonlinearity. When we consider an arbitrary adjustable static nonlinearity, the maximum SNR gain is achieved by a locally optimal nonlinearity for a weak periodic signal in additive white noise [29], [58]. Since the SNR gain of a locally optimal nonlinearity is given by the Fisher information of the noise distribution, we demonstrated that the SNR gain of a locally optimal nonlinearity certainly exceeds unity for a weak periodic signal in additive non-Gaussian noise, and SR does not exist in an updated locally optimal nonlinearity [58]. However, the structure of the locally optimal nonlinearity is determined by the noise probability density function (PDF) and also the noise level [58], [59]. Then, in some practical signal processing tasks, the locally optimal nonlinearity may be too complex to be implemented, and also can not be established for an unknown noise distribution [59]. Therefore, this provides an opportunity for the suboptimal nonlinearity to improve the SNR gain by the SR effect [3], [4], [7], [8], [25]–[29], [58].

In this paper, we focus on amplifying the output-input SNR gain of an uncoupled parallel array of static nonlinearities for transmitting a weak periodic signal in additive white noise. For an array of arbitrary static nonlinearities, the asymptotic expression of the SNR gain is first developed. Then, for a given nonlinearity and fixed noise levels, we prove that the SNR gain of an array is a monotonically increasing function of the array size. It is shown that the SNR gain maximized by the locally optimal nonlinearity is the upper bound of the performance of an array of static nonlinearities. Furthermore, it is demonstrated that the internal array noise components are incapable of further improving the SNR gain for locally optimal processing. This result extends the study of SR in a single static nonlinearity [58], [60] to an array of static nonlinearities. The establishment of a locally optimal nonlinearity needs the complete descriptions of the noise PDF and the noise level. Therefore, when this is not feasible, we propose instead a parallel array of suboptimal but easily implemented threshold nonlinearities for transmitting a weak periodic signal, in order to improve the SNR gain via the SR phenomenon. It is shown that such an array of threshold nonlinearities exhibits the SR effect by increasing the array noise level and the array size. Moreover, with a sufficiently large array size, the fact of the SNR gain exceeding unity is shown for a wide range of underlying noise distributions. These interesting results demonstrate that a parallel array of threshold nonlinearities can be practically exploited, and is useful for nonlinear signal processing.

## Results

### Model

Consider the observation of a process 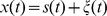, where the component 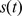 is a weak periodic signal with a maximal amplitude 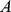 (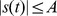) and period 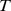, and zero-mean additive white noise 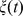, independent of 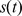, having a PDF 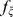 and variance 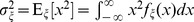. Next, the input 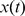 is applied to an uncoupled parallel array of 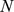 identical static nonlinearities. In these nonlinearities, the noise terms 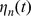, independent of 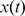, are the internal noise components for each static nonlinearity 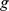, so as to yield the outputs [26].
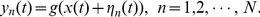
(1)


Here, assume that the derivative 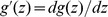 exists for almost all 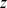, and 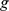 has zero mean under 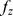, i.e. 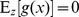, which is not restrictive since any arbitrary 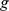 can always include a constant bias to cancel this average [59]. The internal noise components 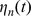 are mutually independent and identically distributed (i.i.d.) with the same PDF 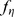 and variance 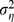. The noise components 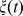 and 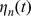 are all assumed to be stationary random variables. Since 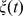 and 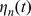 are independent, Eq. (1) can be rewritten as 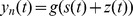, where the composite noise components 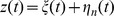 are with the same convolved PDF 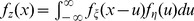. Then, the array output 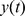 is given by
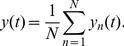
(2)


The input SNR for 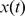 can be defined as the power contained in the spectral line at 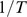 divided by the power contained in the noise background in a small frequency bin 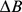 around 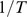, this is [3]

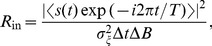
(3)with 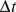 indicating the time resolution or the sampling period in a discrete-time implementation and the temporal average defined as 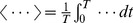 [3]. Since 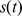 is periodic, 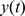 is in general a cyclostationary random signal with period 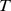 [3]. Similarly, the output SNR at 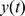 is expressed as
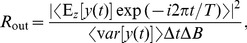
(4)where the nonstationary expectation 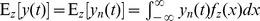 and nonstationary variance 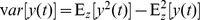 are also temporal functions of time 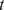 [3]. Then, the SNR gain, 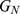, is defined as the ratio of the output SNR over the input SNR [3], [26], [57]

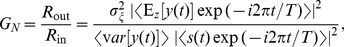
(5)for an array of static nonlinearities with array size 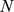.

### SNR Gain of an Array for Weak Signals

For a weak signal 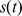 (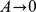 and 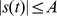) and at a fixed time 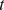, we make a Taylor expansion of 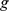 around 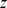 and have the asymptotic form
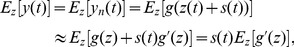
(6)where the outputs 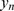 are i.i.d. for 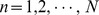. The output degree-two moment is given by
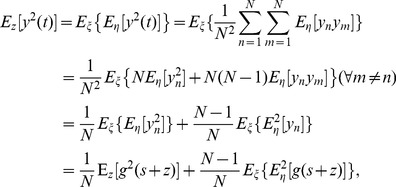
(7)where 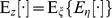 and 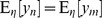. Therefore, based on Eq. (6), we have
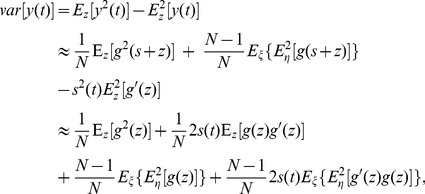
(8)where the approximations are up to first order in the small signal 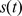. Substituting the asymptotic forms of 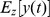 of Eq. (6) and 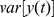 of Eq. (8) into Eq. (5), we obtain the asymptotic expression of the SNR gain of a parallel array of static nonlinearities as
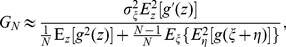
(9)where terms 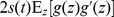 and 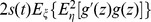, compared with primary terms 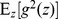 and 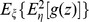, are neglected as 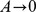 (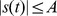). It is interesting to note that the SNR gain 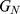 in Eq. (9) is applicable for an arbitrary weak-periodic signal 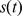 throughout an array of static (yet arbitrary) nonlinearities.

For the random variable 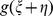 and the convex function 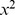, by the Jensen inequality [61], we have
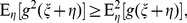
(10)for any fixed variable 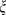 [61]. Therefore, we have



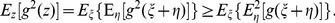
(11)From Eq. (11) and for any integers 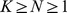, we have
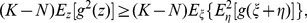
(12)

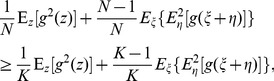
(13)

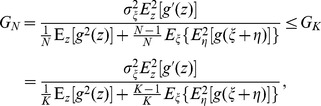
(14)


Thus, for the given nonlinearity 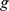 and fixed noise components 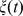 and 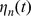, the SNR gain 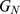 in Eq. (9) is a monotonically increasing function of the array size 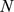. From Eq. (9), we have the minimum.
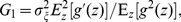
(15)for 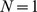, and the maximum
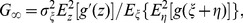
(16)for 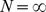.

Naturally, Eq. (12) inspires us to consider the increase of array size 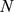 for the further improvement of the SNR gain obtained by a single nonlinearity. We will demonstrate in Eq. (17) that this thought is infeasible for locally optimal processing.

Without the internal noise 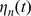, Eq. (9) becomes
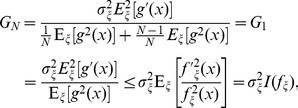
(17)where the array size 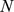 does not work, and the equality occurs as 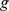 becomes a locally optimal nonlinearity
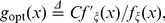
(18)for the derivative 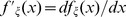 (without loss of generality 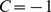) [29], [59]. Here, 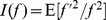 is the Fisher information of the noise distribution 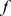 [61].

We add the extra noise 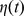 to the observation data 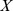, aiming to improve the performance of 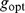. However, based on the Fisher information in inequality 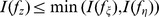 [61], we have
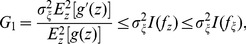
(19)where the later inequality indicates that the addition of extra noise cannot improve the performance of a single locally optimal nonlinearity 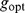 [58].

Based on Eq. (12), the SNR gain of an array of arbitrary static nonlinearities attains its maximum 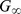 in Eq. (16). Using the Cauchy-Schwarz inequality, we have
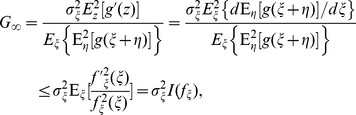
(20)where the nonlinearity 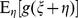 is a function of 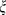. The equality occurs as the nonlinearity 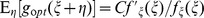, i.e. the nonlinearity 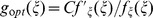 and the PDF 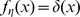. Here, 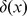 is the Dirac delta function, and this means there is no internal noise in the nonlinearity. From Eqs. (17), (19) and (20), this result indicates that the upper bound of the SNR gain 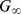 is achieved by 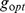 in Eq. (18) without the internal noise 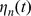. Therefore, the addition of internal noise components 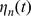 to the signal is never helpful for improving the SNR gain that is obtained by the locally optimal nonlinearity of Eq. (18).

Thus, Eq. (17) extends our previous result of the incapability of SR in the SNR gain improvement of a single locally optimal nonlinearity [58] to the configuration of the array of static nonlinearities. For instance, consider the Gaussian noise components 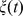 and 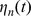 with PDFs 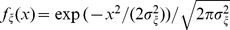 and 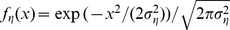, respectively. Then, the composite noise 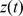 is also Gaussian distributed with PDF 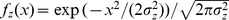 and variance 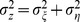. The Gaussian distribution corresponds to the locally optimal nonlinearity 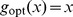 [58]. Substituting 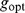 into Eq. (9), we have
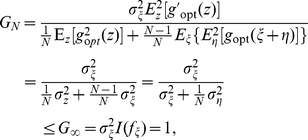
(21)where 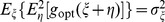 and the Fisher information of 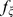 is 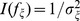 [59]. In Eq. (21), it is seen that the increase of noise variance 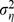 only degrades the SNR gain. The upper bound of unity can be only achieved for the infinite array size 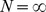 or 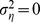 by the locally optimal nonlinearity of Eq. (18).

### Noise-enhanced Signal Transmission in Arrays

It is seen in Eq. (17) that, for transmitting a weak periodic signal in additive white noise, the addition of internal array noise to an uncoupled parallel array of nonlinearities is incapable of improving the SNR gain of the locally optimal nonlinearity 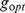. However, the structure of 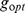 in Eq. (18) depends on the complete description of the noise PDF and the noise level, and in practice it may be difficult to obtain an explicit analytical expression of 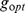 in the unknown noisy environment [59]. Moreover, the presence of internal noise 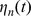 is unavoidable in some practical signal processing cases [5], [6], [38]–[41], [59]. Thus, we place suboptimal but easily implemented nonlinearities in a parallel array to transmit a weak periodic signal, and then show the feasibility of the SR phenomenon [1], [3], [26].

**Figure 1:**
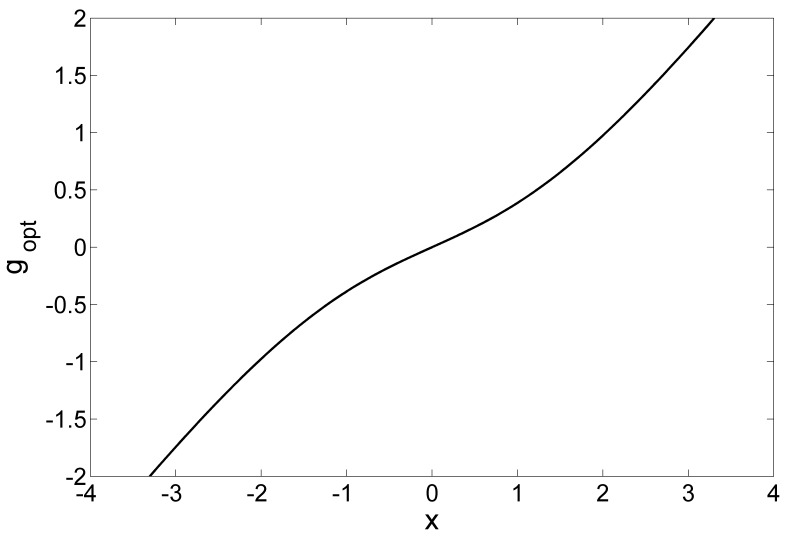
The locally optimum nonlinearity 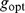. The locally optimum nonlinearity 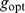 in Eq. (24). The internal uniform noise components 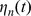 have the RMS amplitude 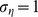. The external noise 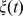 is with the RMS amplitude 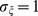 and the decay parameter 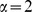 (Gaussian noise).

In the observation model of Eq. (1), the external noise 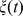 is considered as zero-mean generalized Gaussian noise, which is a flexible family containing some common important cases (e.g. Gaussian noise and Laplacian noise) [27], [59], [61]. The generalized Gaussian noise 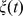 has PDF
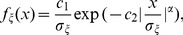
(22)where 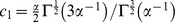 and 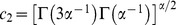 for a decay exponent 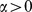 [59]. The array noise terms 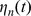 are assumed to be i.i.d. uniform noise with the same PDF
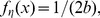
(23)for 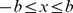 (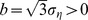) and zero otherwise. When the exponent 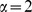, Eq. (22) represents the PDF of Gaussian noise 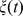. In this case, the signal 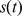 is buried in the composite noise 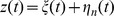. Then, the corresponding locally optimum nonlinearity 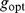 of Eq. (18) needs to be updated as
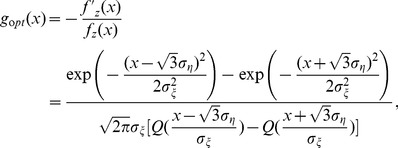
(24)with 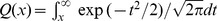. It is seen in Eq. (24) that the structure of 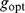 is rather complicated and depends closely on the noise root-mean-square (RMS) amplitudes 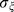 and 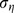. An illustrative plot of the locally optimum nonlinearity is shown in Fig. 1 for 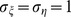.

**Figure 2:**
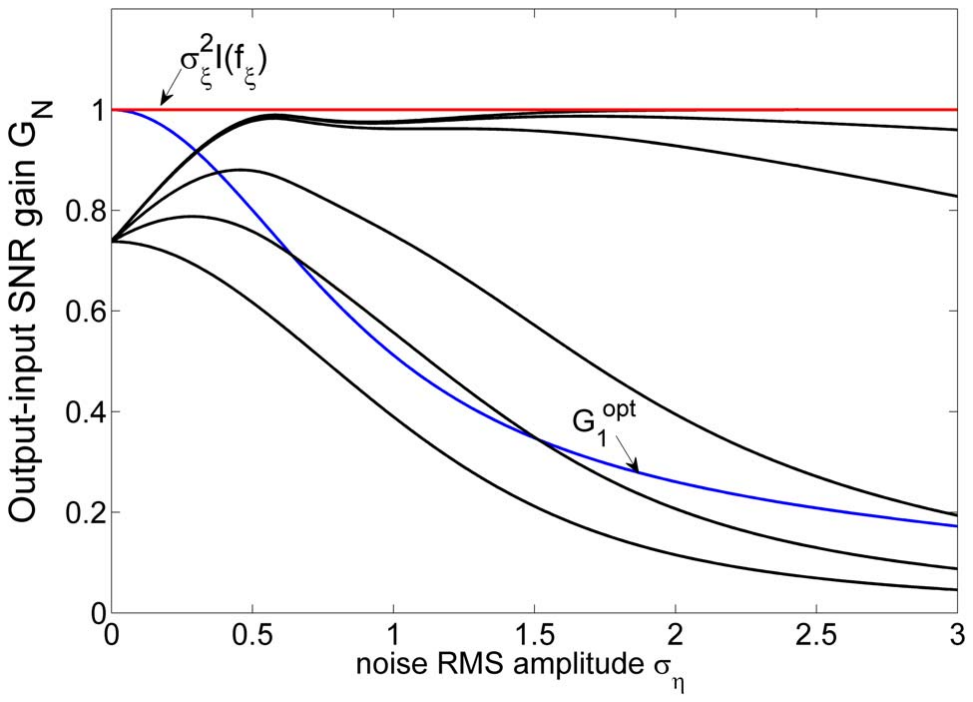
Output-input SNR gain 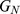. Output-input SNR gain 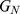 as a function of the RMS amplitude 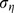 of the array uniform noise terms 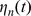 in the array of threshold nonlinearities of Eq. (25). The external noise 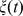 is with the RMS amplitude 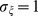 and the decay parameter 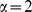 (Gaussian noise). The threshold of 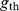 takes 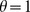. The SNR gain 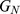 of Eq. (9) is plotted by black lines for 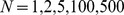 and 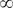 (from the bottom up). For comparison, the SNR gains 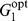 (blue line) of the locally optimum nonlinearities 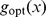 in Eq. (24) and the quantity of 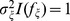 (red line) achieved by the locally optimal nonlinearity 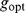 in Eq. (18) (without the internal noise 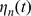) are also illustrated.

A suboptimal but easily implemented nonlinearity that we consider is the three-level threshold nonlinearity [3], [55]

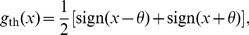
(25)with the sign or signum function 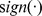 and the response threshold 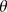. Furthermore, the SNR gain 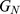 of a parallel array of threshold elements is plotted in Fig. 2 as a function of the RMS amplitude 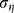 of the array noise 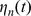 and the array size 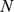. Here, the Gaussian noise 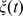 is with RMS amplitude 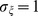, and the response threshold of 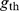 takes 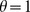. From the bottom up, the SNR gain 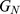 is shown for 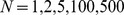 and 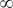 in Fig. 2 (solid lines). It is seen in Fig. 2 that, for an isolated static nonlinearity 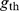 (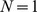), the SR effect does not appear, and the SNR gain decreases monotonically as 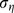 increases. The SNR gain 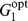 of a single locally optimum nonlinearity 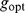 is also plotted in Fig. 2 (dashed line). It is seen in Fig. 2 that the SNR gain 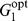 of 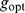 is always better than 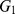 of a single threshold nonlinearity 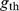 (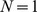). However, as the array size 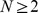 and the array noise RMS amplitude 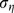 increases, 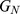 of the array of threshold nonlinearities gradually catches up, and finally exceeds 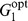 of the isolated locally optimum nonlinearity 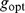, as shown in Fig. 2. Additionally, upon increasing the array size 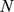, the bell-shape behavior of 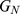 of a parallel array of threshold elements versus 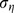 and 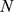 is clearly visible, this is the array SR effect. It is also noted in Fig. 2 that, for a sufficiently large array size 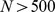, the SNR gain 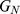 tends to its upper limit of 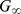 for 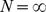. Of course, based on Eq. (17), the upper limit of 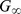 is less than the quantity of 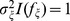 achieved by the locally optimal nonlinearity 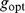 in Eq. (18) (without the internal noise 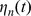), as shown in Fig. 2.

**Figure 3:**
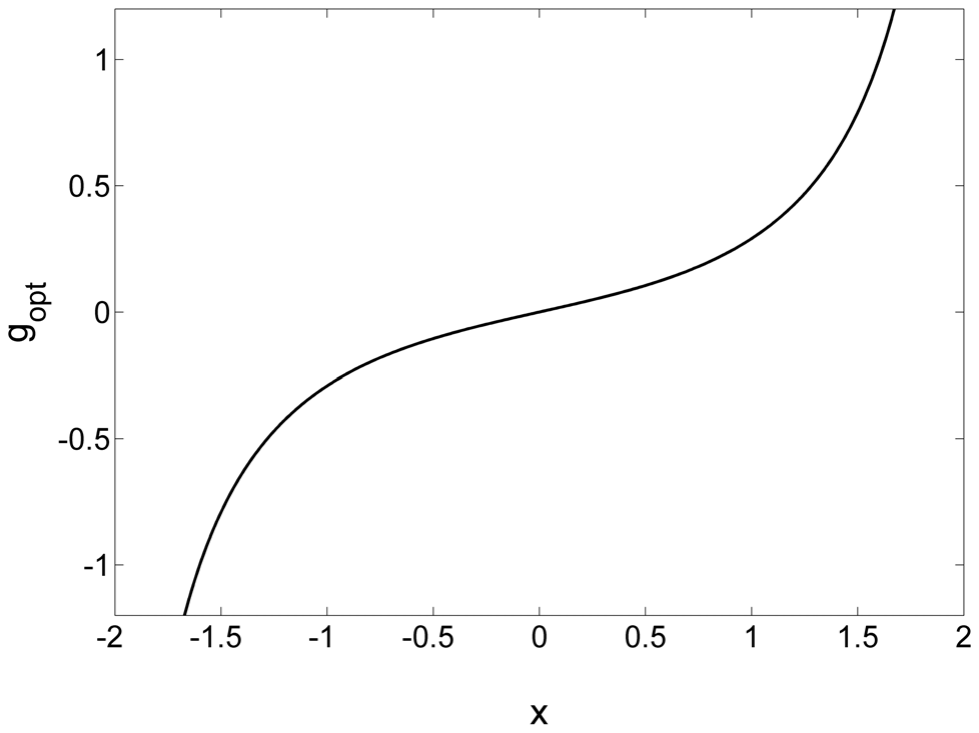
The locally optimum nonlinearity 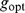. The locally optimum nonlinearity 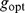 in Eq. (26). The internal noise terms 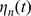 are uniform noises with the RMS amplitude 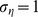. The external noise 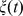 is with the RMS amplitude 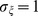 and the decay parameter 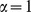 (Laplacian noise).

**Figure 4:**
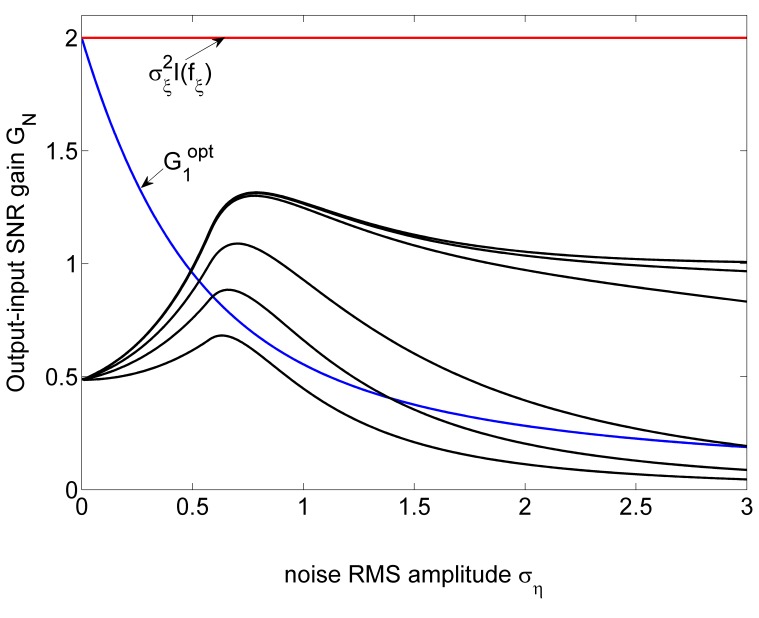
Output-input SNR gain 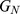. Output-input SNR gain 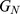 as a function of the RMS amplitude 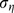 of the array uniform noises 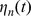 in the array of threshold nonlinearities of Eq. (25). The external noise 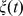 is with the RMS amplitude 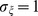 and the decay parameter 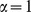 (Laplacian noise). The threshold of 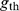 takes 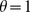. The SNR gain 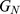 of Eq. (9) is plotted by black lines for 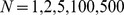 and 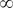 (from the bottom up). For comparison, the SNR gains 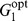 (blue line) of the locally optimum nonlinearities 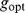 in Eq. (26) and the quantity of 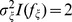 (red line) achieved by the locally optimal nonlinearity 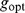 in Eq. (18) (without the internal noise 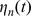) are also illustrated.

Next, an interesting question is, for transmitting a weak periodic signal, whether the SNR gain 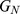 of an array of threshold nonlinearities can exceed unity or not. This possibility, for the case of SNR gain exceeding unity, is shown for Laplacian noise 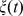 with 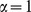 in Eq. (22). In this case, when the array noise components 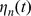 are i.i.d. uniform random variables, the locally optimum nonlinearity should be updated as
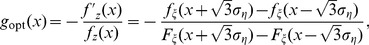
(26)where 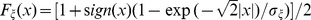 is the cumulative distribution function of Laplacian noise 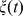. For the noise RMS amplitudes 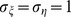, an illustrative example of the structure of 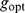 is plotted in Fig. 3. Furthermore, when we fix 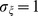 and tune 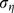, the SNR gains 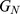 of an array of threshold elements with the threshold 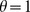 are presented in Fig. 4. It is seen in Fig. 4 that, for a sufficiently larger array size 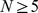, the fact of the SNR gain 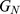 exceeding unity is clearly demonstrated in this case. It is also interesting to note in Fig. 4 that SR effect survives for a single threshold nonlinearity 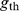 with 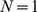.

In Eq. (12), it is known that the performance of an array of nonlinearities increases monotonically with the array size. As indicated in Figs. 2 and 4, we advocate the significance of a parallel array of nonlinearities with large array size 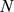: The region of the noise level that improves the SNR gain of an array is gradually expanded as the array size 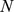 increases. Thus, increasing the array size 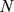 provides a simple alternative means of improving the performance of nonlinearities, especially when the optimal noise associated with a single nonlinearity [28], [30] is not known or accessible.

**Figure 5:**
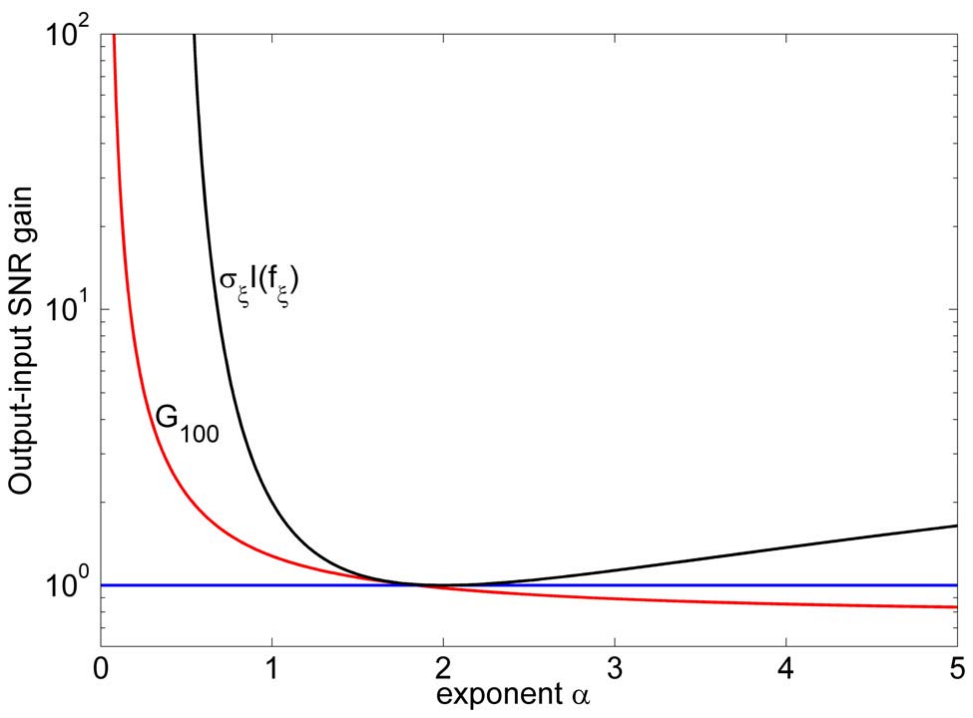
Output-input SNR gain 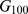. Output-input SNR gain 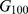 (red line) of an array of threshold nonlinearities with array size 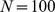 as a function of the decay exponent 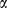 for the response threshold 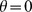. The external noise 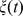 is the generalized Gaussian noise with PDF in Eq. (22) and RMS amplitude 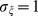. The uniform array noise components 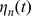 are with the RMS amplitude 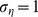. The black line indicates the SNR gain 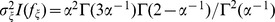 achieved by the locally optimal nonlinearity 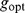 in Eq. (18) (without the internal noise 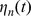), which is also the upper limit that the SNR gain 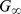 cannot exceed. The blue line is the benchmark of unity.

We also emphasize that the fact of the SNR gain exceeding unity is not exceptive. Here, we employ an array of 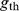 threshold elements with the threshold 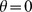 and the array size 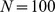. The external generalized Gaussian noise 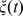 is with the RMS amplitude 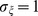. The array noise is uniform noise with its RMS amplitude 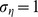. It is shown in Fig. 5 that, for a sufficiently large array size 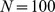, the SNR gain 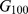 (red line) can be larger than unity for the decay exponent 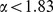, which represents a wide range of generalized Gaussian noise distributions. Here, the black line indicates the upper limit of 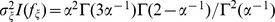 [58], [59] that the SNR gain 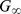 cannot exceed, as Eq. (20) indicated.

## Discussion

In this paper, for a weak periodic signal in additive white noise, we study the characteristics of the SNR gain of an uncoupled parallel array of arbitrary static nonlinearities. Under the assumption of weak signal, an explicit expression of the SNR gain of an array is developed. Then, it is proven that, for a given nonlinearity and fixed noise levels, the SNR gain of an array is a monotonically increasing function of the array size. Furthermore, it is demonstrated that the internal array noise components are incapable of further improving the SNR gain of locally optimal processing. However, since the locally optimal nonlinearity requires a complete knowledge of the underlying noise statics, the structure of the locally optimal nonlinearity may have no analytical expression or be intractable. Therefore, a parallel array of suboptimal but easily implemented threshold nonlinearities becomes an optional approach. It is shown that such an array of threshold nonlinearities can exhibit the SR effect by increasing the array noise level and the array size. For a sufficiently large array size, we also show that the SNR gain of an array of threshold nonlinearities can exceed unity for a wide range of noise distributions, e.g. the exponent 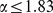 in Fig. 5.

Some interesting open questions arise. For example, we only considered the array of threshold nonlinearities for processing a weak noisy signal. Therefore, can other tractable nonlinearities be connected in parallel for achieving improved output-input SNR gain via the array SR effect? As indicated in Fig. 2 and 4, we can operate an array of nonlinearities with large array size at a feasible level of noise. Therefore, given an acceptance criterion of the performance of nonlinearities, how large the array size is and which level the noise takes are interesting questions. These questions will be of interest for further studies of nonlinear signal processing in the context of array SR, especially in the ensemble of neurons. Often quite a number of neurons have similar properties and respond to the same stimuli [5], [6], [15], thus the condition of all neurons in parallel having the same pattern of input and output connections will be considered. It is of interest to explore how the external (internal) noise components assist the information transfer through the neural network. For the static nonlinearity considered in Eq. (1), Eq. (20) provides the upper bound of the performance of the array of static nonlinearities. While many neuron models, such as the leaky integrate-and-fire model and the Hodgkin-Huxley model [6], [8], represent the neurodynamics with the time evolution nonlinear process (not a static nonlinearity), then whether the transmission efficiency of a parallel array of neurons has an upper bound for the neural signal propagate or not deserves to be studied.

## Methods

Under the assumption of weak signals, the Taylor expansion of the noise PDF is utilized in Eqs. (6), (7), (8) and (9). The Jensen inequality is applied to Eq. (11). The Cauchy-Schwarz inequality is extensively used in Eqs. (17), (20) and (21).
